# Development of the ROOTS Intervention: A Culturally Relevant Parenting Program for Promoting the Emotional and Physical Health of Young Children

**DOI:** 10.1002/jcop.70128

**Published:** 2026-07-13

**Authors:** Daniel K. Cooper, Francesca Lupini, Subina Saini, Guillermo Wippold, Jayxa K. Alonzo, Darha M. Ponder, Ronald J. Prinz

**Affiliations:** ^1^ Department of Psychology and the Research Center for Child Well‐Being University of South Carolina Columbia South Carolina USA

## Abstract

This paper describes the development of a culturally relevant parenting intervention (ROOTS) using the Intervention Mapping (IM) framework. ROOTS was developed through IM's six‐step process by integrating theory, empirical evidence, and community input. A needs assessment identified determinants of child health to guide the Logic Model of Child Health (Step 1), and change objectives were defined in the Logic Model of Change (Step 2). Intervention theory, methods, and practical strategies were selected based on these models and culturally adapted using the Ecological Validity Model (Steps 3–4). Implementation and evaluation plans were developed using a hybrid effectiveness‐implementation approach (Steps 5–6). The final ROOTS intervention includes 6 weekly online sessions emphasizing positive parenting, ethnic‐racial socialization, and healthy lifestyle behaviors. This study demonstrates how IM can be applied to create culturally responsive, dual‐focused interventions that promote feasibility, scalability, and health equity for minoritized families.

In the United States, children from ethnically and racially minoritized backgrounds demonstrate increased risk for adverse outcomes in both *emotional health* (i.e., social, emotional, and cognitive‐behavioral functioning) and *physical health* (i.e., healthy lifestyle behaviors such as sleep and physical activity, and related health indicators such as weight/BMI, chronic illness, and somatic issues) due to the well‐documented impacts of racism (Alegria et al. 2010; Berry et al. 2021; Gómez 2015; Williams et al. 2019). Historical and ongoing manifestations of *structural racism* have contributed to persistent inequitable access to safe housing (e.g., racial residential segregation), high‐quality education and healthcare, and healthy food (Williams et al. 2019). *Interpersonal racism*, including daily experiences of discrimination, further contributes to the increased rates of health problems among minoritized children, especially Black and Latiné children (Berry et al. 2021; Williams et al. 2019). Structural and interpersonal racism are associated with adverse health outcomes, including chronic stress, emotional dysregulation, and poor sleep (Mayne et al. 2021; Williams et al. 2019). Considering their disproportionate exposure to these forms of racism, it is not surprising that Black and Latiné children often demonstrate higher rates of obesity, shorter sleep duration, and increased internalizing symptoms compared to their White counterparts (Alegria et al. 2010; Berry et al. 2021; From Crisis to Opportunity: Reforming Our Nation's Policies to Help All Children Grow Up Healthy 2021; Lupini and Williamson 2023).

These disparities can be understood through a community psychology lens, which emphasizes prevention, health promotion, and the role of social and structural contexts in shaping individual and family well‐being (Fairweather 1980; Kloos et al. 2021). From this perspective, child health disparities are not solely a function of individual behavior but reflect multilevel influences, including systemic inequities, community environments, and access to resources (Munger et al. 2016; Rappaport 1977). Accordingly, interventions are assumed to be most effective when they adopt an ecological perspective, target modifiable family‐level processes, and remain responsive to the broader sociocultural context in which families are embedded (Trickett 2009), highlighting the need for approaches that are both culturally grounded and capable of addressing multiple, interrelated domains of child health.

In light of these inequities, there is a pressing need for culturally responsive, *dual‐focused interventions* that target both emotional and physical health. A dual‐focused approach is warranted because children's emotional and physical health share overlapping behavioral and environmental determinants (e.g., exposure to racial stressors, effective parenting practices). The traditional approach of delivering multiple, separate programs is often more costly and time‐consuming, and may result in repeated content. In contrast, a single curriculum that targets shared mechanisms can reduce burden and improve feasibility for families and providers, while also capitalizing on synergistic or cross‐domain effects (Hale et al. 2023). Moreover, previous research demonstrates that racially and ethnically minoritized families are less likely to engage in services due to increased barriers (Dickson et al. 2017). As such, optimizing a single intervention to address both domains of child health may increase benefits gained, improve access to resources, and minimize burden on families experiencing multiple barriers.

Parenting interventions may be particularly well‐suited to address children's emotional and physical health simultaneously. From a community psychology perspective, parenting interventions represent a key preventive strategy for promoting population‐level health by targeting modifiable family processes that influence child development (Trickett 2009). In fact, a large body of research demonstrates that positive parenting practices are one of the most critical and modifiable mechanisms to support child health (Shonkoff 2000). Positive parenting is characterized by warm, supportive parenting practices such as effective communication, responsiveness, positive reinforcement, parental monitoring, and consistent and appropriate discipline, all of which have been linked to greater child emotional health (e.g., greater prosocial behaviors, fewer internalizing and externalizing behaviors) and physical health (e.g., sleep, physical activity, and eating habits; Ling et al. 2017; Ochoa and Berge 2017; Pinquart 2017; Ward et al. 2023). Further, several parenting interventions that promote positive parenting have demonstrated efficacy in improving children's emotional and physical health. For instance, meta‐analyses examining The Incredible Years and Triple P‐Positive Parenting Program, two of the most widely disseminated parenting programs, indicate that implementation of positive parenting practice improves child social (e.g., prosocial behaviors), emotional (e.g., anxiety), and behavioral (e.g., disruptive behaviors) outcomes (Menting et al. 2013; Sanders et al. 2014). Another study examining the effects of an evidence‐based parenting intervention found that participation led to improvements in body mass index (BMI) trajectories across childhood (Smith et al. 2015). Despite the growing evidence for parenting as a mechanism of change in both domains of health, few parenting interventions have targeted emotional and physical health simultaneously.

In addition to a dual focus on both domains of health, it is crucial for parenting interventions to incorporate culturally relevant strategies, particularly when designed for minoritized families. For instance, ethnic‐racial socialization is an important cultural process that has been linked to a host of positive child health outcomes among minoritized families (Umaña‐Taylor and Hill 2020). Ethnic‐racial socialization refers to how families transmit information about culture, including values, traditions, and practices, and may serve as a key protective factor in buffering the impact of racism on children's emotional health, physical health, and healthy lifestyle behaviors (Brody et al. 2021; Conway et al. 2021; Granberg et al. 2009; Neblett et al. 2008; Wang et al. 2022). Two of the ethnic‐racial socialization practices most consistently linked with positive child outcomes are fostering racial or ethnic pride (also known as *cultural socialization*) and increasing children's awareness of and preparation for coping with discrimination (*preparation for bias*). Since the 1980s, when the concept was first presented in the literature (Peters and Massey 1983), studies have shown that promoting pride contributes to a positive ethnic‐racial identity and a sense of belonging (Stevenson and Arrington 2009), which are linked to higher self‐esteem and fewer internalizing symptoms (Mandara et al. 2009; McHale et al. 2006; Umaña‐Taylor et al. 2009). Preparation for bias messages may be linked to greater use of racial coping skills, reducing uncontrollable threat appraisals and stress reactivity (Anderson et al. 2018; Anderson and Stevenson 2019). Ethnic‐racial socialization is also associated with greater parent‐child warmth and communication (Frabutt et al. 2002; McHale et al. 2006), which can help foster emotion regulation and healthy lifestyle behaviors (Ray et al. 2013; Richardson et al. 2023). For example, Black adolescents whose parents engaged in more ethnic‐racial socialization during childhood experienced fewer increases in sleep problems during adolescence (Conway et al. 2021). Another study using data from two family‐based interventions that integrated ethnic‐racial socialization with Black families concluded that the intervention successfully buffered the impact of discrimination on multiple child outcomes, including conduct problems, depression, and anxiety symptoms (Brody et al. 2021).

Despite the growing evidence that ethnic‐racial socialization may promote children's health and mitigate the harmful effects of racism, few parenting interventions have integrated this key factor. For example, of the 36 family‐based interventions listed on the Blueprints for Healthy Youth Development registry, only one included content on ethnic‐racial socialization (*Blueprints for Healthy Youth Development – Committed to Healthy Youth, Families and Communities* n.d.; Cooper et al. 2025). As such, the inclusion of ethnic‐racial socialization remains a critical gap in the family‐based intervention literature that may limit minoritized families’ engagement in and trust of parenting programs. Further, Black families report ethnic‐racial socialization as a critical aspect of raising children; therefore, honoring the cultural strengths of families of color within parenting programs may increase engagement, buy‐in, and benefits (Coard et al. 2004). Emphasizing cultural strengths is an important aspect of community psychology and remains underutilized in family‐based prevention (Buckley et al. 2025).

## Intervention Mapping as a Framework for Developing Evidence‐Based Health Programs

Developing culturally responsive, dual‐focused interventions is a complex process that requires balancing multiple theoretical, empirical, and cultural considerations. The Intervention Mapping (IM) framework provides a systematic, theory‐ and evidence‐based approach to navigating these challenges across the design, implementation, and evaluation phases of program development (Fernandez et al. 2019). It integrates behavioral and social science theories and empirical data to create programs that are effective and tailored to the specific needs of a target population, which is especially important when creating multi‐component interventions for culturally diverse populations. The IM process comprises six iterative steps: (1) assessing the target health issue along with its behavioral and environmental determinants; (2) defining desired behavioral and environmental outcomes and formulating specific change objectives; (3) selecting theory and evidence‐based behavior change methods and translating them into practical strategies; (4) organizing program components into a coherent intervention; (5) planning for adoption, implementation, and sustainability; and (6) developing comprehensive process and outcome evaluation plans (Fernandez et al. 2019).

The IM framework has been applied to various contexts, including workplace health promotion, school‐based interventions, and community health programs, with many applications involving disease management in clinical or medical settings. In Finland, the DAGIS intervention used IM to design a preschool‐based and family‐based program that promoted healthy eating, physical activity, and self‐regulation in young children (Ray et al. 2019). Researchers developed culturally appropriate educational materials and family activities that were practical, low‐cost, and easy to integrate into daily routines. The resulting 22‐week program provided coordinated resources for preschools and families, targeting multiple health behaviors across diverse populations. Similarly, the MINDSET project applied IM to develop a mobile health application designed to support self‐management among adults with epilepsy (Shegog and Begley 2017). This intervention focused on four key areas: medication adherence, seizure response, trigger avoidance, and management of co‐occurring health conditions. The IM framework provided structured guidance throughout the development process, from collaborative planning and stakeholder input to content development, prototype creation, and planning for implementation and evaluation (Shegog and Begley 2017).

These examples demonstrate IM's flexibility and effectiveness for creating interventions that target complex health behaviors in different populations. However, despite the growing use of IM, few studies have used it to develop family‐based interventions that address both emotional and physical health outcomes in young children, or that explicitly integrate cultural strengths such as ethnic‐racial socialization. One of the few such studies used IM to develop a childhood obesity prevention program focused on pregnant women and new mothers, integrating components of healthy eating, physical activity, and infant feeding practices (Taylor et al. 2013). This intervention was specifically designed for a diverse community, addressing cultural barriers and incorporating perspectives from White and South Asian families. While this example illustrates IM's utility for adapting interventions to diverse families, it remained limited to single‐domain health targets.

### The Present Study

To address the gaps in culturally relevant, dual‐focused interventions for young children, we developed the Resilience‐Oriented Outcomes and Techniques for Stronger Families (ROOTS) program using an IM approach. The ROOTS program weaves positive parenting, ethnic‐racial socialization, and health promotion content into a culturally responsive intervention for Black and Latiné families. To our knowledge, ROOTS is the first dual‐focused parenting intervention to integrate ethnic‐racial socialization as a core component, representing an important contribution to the prevention science literature. In addition, this study demonstrates how principles central to community psychology, including prevention, ecological perspectives, strengths‐based approaches, and attention to structural inequities (Kloos et al. 2021; Rappaport 1977), can be operationalized within a systematic intervention development framework. While most intervention protocols focus primarily on recruitment, measurement, and outcome evaluation, our study centers on the process of intervention development, demystifying the process of designing a culturally relevant, dual‐focused intervention. The research team followed the six IM steps, integrating theory, empirical evidence, and community input to design and evaluate a health promotion program. The following sections outline the methods and results for each IM step, demonstrating how IM can be strategically applied and adapted to develop culturally grounded interventions that simultaneously address multiple health domains.

## 1 Method and Results

### 1.1 Step 1: Understanding the Target Health Outcome and Its Determinants

The first step in the IM framework involves assessing the target health concern, identifying its behavioral and environmental causes (i.e., determinants), and defining the contextual factors that will shape the intervention, including the target population, setting, and delivery format. This process involved three key tasks: (a) conducting a qualitative needs assessment, (b) reviewing local community health reports, and (c) synthesizing empirical research to identify modifiable family‐ and environmental‐level factors influencing child emotional and physical health. Findings from these tasks were integrated into a logic model of child health, which mapped the interaction of these factors and their influence on the targeted health outcomes. While the original IM approach frames this step as the logic model of the problem, we adapted the framing to be more consistent with a strengths‐based, health promotion approach. Past literature with minoritized families is saturated with deficit‐focused conceptualizations of health (Bowleg 2023; Márquez‐Magaña 2024; Wang et al. 2022), which can reinforce negative stereotypes and overlook essential cultural protective factors. Therefore, our adapted model, which we refer to as the “Logic Model of Child Health,” specifies child health as the target outcome (instead of child health problems). This more neutral framing encompasses positive and negative health influences. For example, a child's emotional health consists of prosocial behaviors as well as disruptive or aggressive behaviors. We believe this strength‐oriented approach better aligns with health equity and resilience frameworks (Goodrum et al. 2024; Murry et al. 2018), which underlie our study. The following sections describe the methods used for each of the three Step 1 tasks and summarize key findings that informed the development of our logic model.

#### 1.1.1 Needs Assessment

Our first task consisted of conducting a qualitative needs assessment with local parents and service providers to better understand the determinants of child emotional and physical health in the specific context, as well as their preferences for intervention delivery. Needs assessments are a critical component of program development, providing a structured approach to identifying gaps between current conditions and desired outcomes and informing the design of socioculturally relevant interventions (Kettner et al. 2022; White and Altschuld 2012). Best practices emphasize incorporating input from community members and service providers to ensure that programs reflect community priorities, strengths, and lived experiences, with the ultimate goal of enhancing program engagement and impact (Small et al. 2009).

Consistent with these principles and a strengths‐based approach, we explored community perspectives on the assets parents drew upon to promote their children's health as well as the challenges they faced. We interviewed 33 parents and six providers (e.g., community health workers, school counselors) who worked closely with Black and Latiné families. Participants emphasized several key parenting priorities, including supporting children's social‐emotional development, promoting healthy eating, maintaining good sleep hygiene, encouraging physical activity, and managing screen time. Many also underscored the importance of culturally relevant parenting practices (e.g., ethnic‐racial socialization), especially strategies that foster ethnic‐racial pride and prepare children to navigate discrimination. Parents expressed a desire for culturally relevant parenting support that integrates positive parenting, ethnic‐racial socialization, and healthy lifestyle promotion into a cohesive program. They also indicated a preference for a brief, virtually delivered format led by culturally matched facilitators (see Cooper et al. 2024; Lupini et al. 2025 for additional details).

### 1.2 Local Community Health Reports

The second task involved corroborating and extending the needs assessment findings by reviewing local health data sources, such as a statewide needs assessment that involved over 5000 parents, service providers, and researchers (South Carolina Department of Human Servicers 2019). The study used a mixed‐methods approach, incorporating structured working group meetings, online surveys, and focus groups to identify priority areas for early childhood health promotion. Parents reported that the most pressing needs for supporting child health were (a) increased understanding of child development and (b) access to affordable family health services. Other important priorities included spending quality time with their children, maintaining strong parent‐child relationships, and having a supportive network of friends and family. These themes mirrored many of those from our qualitative interviews and underscored the need for early access to parenting support and family health resources.

#### 1.2.1 Literature Review

The third task was to conduct an informal literature review on parenting and child health promotion. Overall, findings from the needs assessment and health reports aligned with a well‐established body of empirical research on child health determinants. One of the most widely supported frameworks in this area is the Family Stress Model, which posits that environmental stressors, such as poverty, community violence, structural disadvantage, and discrimination, undermine parenting practices and disrupt child development (Masarik and Conger 2017). Numerous studies demonstrate that higher levels of positive parenting (e.g., effective communication, positive reinforcement, consistent discipline) and lower levels of harsh parenting (e.g., shouting, hitting) are associated with better emotional regulation, increased prosocial behavior, and healthier lifestyle behaviors in children, including improved sleep, nutrition, and physical activity (Collins et al. 2014; Pinquart 2017; Ward et al. 2023).

Cultural adaptations of the family stress model, such as integrative models of cultural resilience (Carlo et al. 2022; Murry et al. 2018), emphasize the role of ethnic‐racial socialization as a key protective parenting process fostering healthy child development. Two strategies (i.e., promotion of pride and preparing children for bias) appear particularly effective in promoting positive developmental outcomes (Umaña‐Taylor and Hill 2020). A recent meta‐analysis found that racial pride‐promoting practices were robust predictors of youth outcomes, including stronger ethnic‐racial identity, higher self‐esteem, greater academic achievement, more adaptive coping, and improved psychological well‐being (Umaña‐Taylor and Hill 2020). Together, these frameworks informed our understanding of the culturally relevant parent and environmental factors that shape child health, which we integrated into our working logic model described below.

#### 1.2.2 Logic Model of Child Health

**Figure 1:**
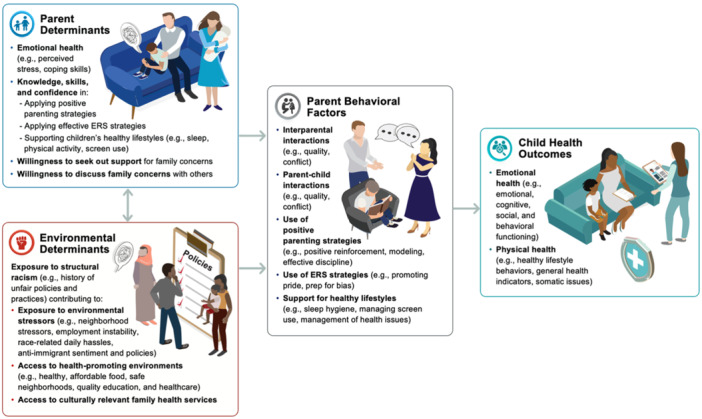
Logic Model of Child Health. ERS = ethnic‐racial socialization.

Findings from the needs assessment, community health reports, and literature review were integrated into a logic model of child health (Figure 1). This approach aligns with best practices for logic model development, which emphasize integrating empirical evidence and contextual data to specify relationships among determinants, behaviors, and outcomes to guide intervention planning (McLaughlin and Jordan 1999; Renger and Hurley 2006). This model identifies how key environmental determinants (e.g., exposure to neighborhood stressors, access to health‐promoting resources) interact with parent determinants (e.g., parents’ emotional functioning, parenting knowledge). These factors, in turn, influence parenting behaviors (e.g., use of positive parenting strategies, support for healthy child routines), ultimately contributing to child emotional and physical health. This model enabled us to differentiate among behaviors, environmental factors, and their underlying influences, allowing us to more clearly define the changes and outcomes to be addressed in Step 2.

### 1.3 Step 2: Determining Performance and Change Objectives

The second step of IM involves developing specific performance and change objectives (i.e., a Logic Model of Change) that, if modified, would lead to improvements in child health. This process built upon the data and results obtained in Step 1 by breaking down the expected behavioral and environmental outcomes into (a) more proximal observable behaviors (i.e., performance objectives) and (b) the determinants that give rise to these behavior changes (i.e., change objectives). Whereas the Logic Model of Child Health in Step 1 focused on describing the range of multi‐level factors (e.g., individual, family, community) associated with the target health outcome, the logic model of change is more focused on the specific, feasible, modifiable factors that can be influenced via intervention. Structural or more distal determinants outside the scope of the intervention (e.g., exposure to structural racism) are not included in the Logic Model of Change. This Logic Model of Change then serves as the foundation for selecting evidence‐based strategies to incorporate into a health promotion intervention in Step 3. Below, we describe and justify each aspect of our Logic Model of Change, starting with our desired outcome, improvements in child health.

The expected child health outcomes included improved emotional health, which we defined as increased emotional, social, cognitive, and behavioral functioning, and improved physical health, defined as healthier lifestyle behaviors and health status indicators. These are the ultimate targets of the intervention, which, if changed at a broad scale, would be expected to decrease population‐level health disparities, improving health equity (DeVoe et al. 2019). We further broke down the child health outcomes into shorter‐term and longer‐term outcomes to better reflect the change process documented in the literature (Monteiro et al. 2005). For example, improving children's health status or disease symptoms (longer‐term outcomes) requires more immediate changes in lifestyle behaviors, such as healthier eating habits or stricter adherence to disease symptom management (shorter‐term; National Resource Center for Health and Safety in Child Care and Early Education 2020). Our assumption is that changes in various parenting behaviors and practices (performance objectives) will lead to changes in child‐level health outcomes (Stormshak et al. 2025), which we describe next.

#### 1.3.1 Performance Objectives

Our performance objectives focused on modifiable parenting behaviors to be targeted in the intervention, which were derived from our needs assessments and literature reviews. These behaviors expanded on the concepts presented in the Logic Model of Child Health, encompassing parent‐child interactions, the use of positive parenting and ethnic‐racial socialization strategies, and support for healthy child routines, all of which influence child health outcomes (Brody et al. 2021; Ling et al. 2017; Smith et al. 2015). For example, we identified specific positive parenting strategies that contribute to child health, such as positive reinforcement, modeling, effective communication, and limit setting (Cooper et al. 2025). These strategies were selected based on their consistent association with child health outcomes and their identification as key ingredients of evidence‐based parenting interventions (Leijten et al. 2019). Additionally, we found that many of the positive parenting strategies overlap with effective strategies for promoting ethnic‐racial socialization and healthy lifestyles (Coard et al. 2004; Stein et al. 2021). For example, positive reinforcement, such as praise, can be used to foster ethnic‐racial pride or encourage healthy sleep habits (Hiscock et al. 2015; Stein et al. 2021). Likewise, limit setting and effective discipline can be used to discourage unwanted behaviors, such as limiting video content that reinforces racial stereotypes or limiting screen use before bedtime (Cooper et al. 2025). This overlap also allows for greater ease in bundling these strategies into an intervention.

#### 1.3.2 Change Objectives

**Figure 2:**
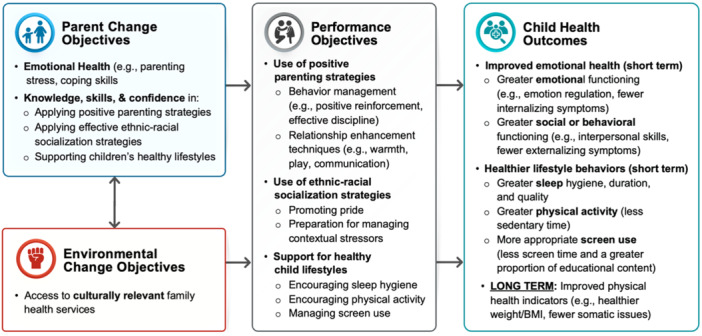
Logic Model of Change.

In order to observe changes in the parenting behaviors identified in our performance objectives, several earlier and multilevel conditions must be addressed. These proximal factors that influence our performance objectives are referred to as change objectives and are separated into parent‐level and environmental‐level objectives. For example, by improving access to high‐quality, culturally responsive parenting support (environmental change objective), we expect positive changes in parents’ knowledge of and skill in applying effective parenting strategies as well as greater ability to manage parenting stressors (parent change objectives). These changes, in turn, are expected to reduce the impact of racial stressors on child health outcomes. These factors were identified in our literature review and have been shown to impact the parent behaviors identified in our performance objectives. The Logic Model of Change is presented in Figure 2.

### 1.4 Steps 3–4: Designing the ROOTS Program

Steps 3 and 4 of IM involve designing and packaging the intervention using the Logic Model of Change as a guide. This process consisted of four main tasks: (a) generating program themes and components, (b) selecting theory and evidence‐based methods, (c) translating those methods into practical intervention strategies, and (d) pretesting and refining the strategies to develop the final program. Each task was informed by the literature on evidence‐based parenting interventions and the results of our needs assessment, which highlighted the importance of a brief, flexible, virtually deliverable program that incorporated content on ethnic‐racial socialization (Lupini et al. 2025).

#### 1.4.1 Task 1: Generating Program Components

We identified positive parenting, ethnic‐racial socialization, and healthy lifestyle promotion as the three major program components. This decision was based on (a) our review of empirical literature (i.e., via a scoping review), (b) practical considerations, such as the feasibility of combining the components into a brief, unified program, (c) culturally relevant risk and resilience frameworks emphasizing the importance of cultural strengths, and (d) findings from our needs assessment. Our scoping review of dual‐focused parenting programs revealed that most successful interventions addressed positive parenting and healthy lifestyle behaviors (Cooper et al. 2025), but none included ethnic‐racial socialization. This presented a unique opportunity for ROOTS to contribute to the literature on culturally relevant, dual‐focused health promotion. In line with prior evidence that simpler prevention programs often yield stronger outcomes (Leijten et al. 2019), we aimed to include only the most essential positive parenting components, leveraging strategies that can be used to jointly impact child emotional and physical health outcomes, while minimizing additional content. Our goal was to apply foundational parenting strategies in ways that jointly promote racial socialization and healthy lifestyle behaviors, utilizing the natural overlap in these strategies to avoid redundancy and burden.

#### 1.4.2 Task 2: Selecting Theory and Evidence‐Based Methods

The second task involves identifying general evidence‐based principles known to support behavior change in the determinants outlined in the Logic Model of Change. These principles can be viewed as the broader conceptual framework that guides the development of specific strategies in the next step. Theory and methods undergirding evidence‐based parenting support are based on principles of operant and social learning theories (Leijten et al. 2019; Stein et al. 2021). These theories posit that learning occurs as a result of social observations, rewards, reinforcements, and consequences, such that behaviors that are rewarded or reinforced are more likely to be repeated, while those followed by negative outcomes or ignored become less frequent (Bandura 1977; Patterson 1982; Skinner 1950). These principles are flexible and can be applied to promote any desired behavior (e.g., prosocial behavior, ethnic‐racial pride, or healthy lifestyle habits). For example, parents can model and praise behaviors that reflect racial pride or healthy lifestyle choices (Hiscock et al. 2015; Stein et al. 2021). Likewise, they can use effective discipline and limit‐setting to reduce sedentary behavior or screen use (Cooper et al. 2025). Additionally, the four existing parent‐based ethnic‐racial socialization interventions also draw from social learning theories, including step‐wise learning, verbal encouragement, and vicarious social models (Anderson et al. 2018; Coard et al. 2007; Murry et al. 2007; Stein et al. 2021). Parenting programs focused on healthy lifestyle promotion also model off these principles (Cooper et al. 2025; Smith et al. 2017), suggesting a shared theoretical foundation across domains.

#### 1.4.3 Task 3: Selecting Practical Strategies

The next task involved translating the theories and methods outlined above into practical strategies. This process consisted of identifying which strategies to include, determining how to sequence and present them coherently, and making sure they were accessible and engaging to our target population. We prioritized instructional methods commonly used in evidence‐based parenting support, such as didactic teaching, role‐play demonstrations, reflective practice, and collaborative goal‐setting, which have demonstrated efficacy across the domains of positive parenting, ethnic‐racial socialization, and healthy lifestyle promotion (Anderson et al. 2018; Prinz 2019; Smith et al. 2017). To select strategies grounded in operant and social learning theories, we drew from the Triple P – positive parenting program due to its (a) well‐established evidence base, (b) use in various populations and contexts, and (c) flexible delivery formats (e.g., online or in‐person, individual or group‐based, brief or intensive). Triple P teaches strategies such as behavior monitoring, positive reinforcement, and effective discipline, skills that align closely with our aims to promote child emotional and behavioral health and the pathways specified in our logic models. These parenting strategies also lend themselves naturally to techniques for promoting ethnic‐racial socialization and healthy lifestyles (as described in Task 2).

To incorporate ethnic‐racial socialization strategies, namely promoting pride and preparation for bias, we drew from the One Talk at a Time intervention (Stein et al. 2021). This intervention shares a theoretical foundation with Triple P and teaches similar techniques, such as praise and effective communication skills. Therefore, we braided ethnic‐racial socialization strategies with the positive parenting strategies used to encourage desired behavior.

We also weaved in content on healthy lifestyle behaviors, beginning in Session 1, where parents discuss the common causes of child misbehavior. Specifically, we introduce how sleep, physical activity, and screen time can impact children's behavior and well‐being. Although some positive parenting programs like Triple P mention how unhealthy lifestyle behaviors (e.g., poor sleep, excessive screen use) can give rise to behavior problems, most do so at a surface level (Cooper et al. 2025). Our program aimed to place greater emphasis on healthy lifestyles, such as by teaching parents the current public health recommendations for sleep, physical activity, and screen time, and encouraging them to reflect on their children's challenges in these areas. In Sessions 2–4, parents apply the positive parenting skills to promote improvements in these lifestyle areas. This sequencing reflects our effort to support parents in applying general parenting strategies to promote children's ethnic‐racial identity development and healthy lifestyle behavior. We created a matrix summarizing how we linked performance objectives to theories and practical strategies (Table 1).

**Table 1:** Linking Performance Objectives to Theories and Practical Strategies.

Performance Objectives	Theories	Practical Strategies
Positive Parenting		
Warmth and positive reinforcement	OSLT	Spending quality time, having interesting activities, using descriptive praise, showing affection, using behavioral charts
Modeling desired behaviors	OSLT	Modeling effective communication and healthy lifestyle behaviors, such as sleep hygiene, limiting screen use, and engaging in family activities
Effective communication	OSLT	Discussing household ground rules, open‐ended questions, giving clear, calm instructions, incidental teaching, Ask‐Say‐Do
Limit setting and effective discipline	OSLT	Practicing parenting routines: giving clear instructions, then backing it up with logical consequences or time out
Ethnic‐Racial Socialization Strategies		
Promoting pride	OSLT	Teaching strengths of their culture (e.g., reading books, celebrating cultural holidays, sharing family recipes), sending positive messages to their child
Preparation for bias	OSLT	Identifying barriers to having challenging conversations, role‐playing how to respond to child when they disclose experiencing discrimination or bullying (use of open‐ended questions, validating child's thoughts and emotions, brainstorming ways to respond)
Support for Healthy Lifestyles		
Encouraging healthy sleep	OSLT	Applying skills for encouraging desired behavior (e.g., praise, Ask‐Say‐Do) and effective discipline to encourage consistent bedtime routines and minimizing bedtime parent‐child conflict
Encouraging physical activity	OSLT	Applying strategies for encouraging desired behavior (e.g., descriptive praise, behavior charts) to promoting healthy physical activity
Managing screen use	OSLT	Applying parenting routines (e.g., giving clear instructions, backing it up with logical consequences and time out) to limit screen use

*Note:* OSLT = Operant and Social Learning Theories, such as step‐wise learning, verbal encouragement, and vicarious social models.

#### 1.4.4 Task 4: Packaging Strategies to Produce the Final Program

Task 4 included packaging the various strategies selected in Task 3 into a final product that is culturally relevant. This involves producing program materials, such as the language, photos, graphics, and video content, as well as pre‐testing the materials to ensure a fit with the given context and population. Our bilingual, multicultural research team worked together to produce materials in English and Spanish, select culturally relevant images, and discuss ways to package the information that were consistent with the values, practices, and norms of English‐ and Spanish‐speaking Black and Latiné families. This adaptation process was guided by the Ecological Validity Model (Bernal et al. 1995), which outlines eight dimensions to consider when adapting content to fit a particular cultural context: language, metaphors, persons, content, concepts, goals, methods, and context. Each dimension is described, along with specific examples in Table 2. We also made several adaptations to better fit the online format, such as allowing families to send in video recordings of their skills practice task instead of doing a live demonstration and using child interruptions as an opportunity to practice limit‐setting skills.

**Table 2:** Cultural Adaptations to Program Content and Delivery.

Topic	Definition	Examples
Language & Metaphors	Ensure that materials and delivery use the appropriate language, dialect, and literacy level, including culturally resonant symbols, sayings, stories, or images to enhance relatability and engagement.	Translated materials into Spanish, adapted images, and incorporated culturally relevant storybooks. Parent coaches, who were native speakers, used culturally appropriate language and metaphors. Used culturally appropriate words (e.g., ‘*tiempo de calma*’ for ‘quiet time’). Selected optimal terms for the parent coach role (coach instead of interventionist, *instructor/a* instead of *facilitador/a*).
Persons	Consider the match between the therapist/facilitator and participants (e.g., cultural background, shared identity).	Matched parent coaches and families by ethnic‐racial background and language preference to promote trust and strengthen shared identity.
Content & Concepts	Tailor examples, values, and cultural knowledge to reflect the group's lived experiences and belief systems. Align psychological constructs with culturally meaningful understandings.	Encouraged parent coaches to use Latiné historical figures and heroes as examples to help parents prepare their children for bias. Selected the term “bullying” instead of “discrimination,” as collaborators found Latiné parents often underreport bias when only the term “discrimination” is used.
Goals	Ensure the goals of the intervention reflect culturally valued outcomes (e.g., community harmony, respect, spirituality).	Allowed parents to set goals for themselves and their child, with flexibility built into the program design.
Methods	Adapt delivery strategies to be culturally appropriate.	Assigned an English or Spanish‐speaking point person (depending on family's preferred language) to each family for ongoing logistic support. Incorporated culturally relevant storybooks. Selected a virtual format based on feedback from parents and providers in qualitative interviews. Included dedicated time for relationship building in Session 1, reflecting the cultural values of *personalismo* and *comunalism*.
Context	Address broader social and structural factors influencing the population (e.g., racism, immigration status, poverty).	Incorporated ethnic‐racial socialization content by selecting books tailored to Black and Latiné families. Implemented role‐play activities to practice difficult conversations about race, culture, and preparing for bias.

### 1.5 Step 5–6: Implementation and Evaluation Planning

Steps 5 and 6 of IM focus on designing strategies to promote the adoption, implementation, and sustainability of an intervention in real‐world settings. These steps include developing a plan for process and outcome evaluations to assess effectiveness, fidelity, adaptations, and overall impact. As outlined in earlier steps, our community needs assessment served not only to identify target outcomes but also to highlight potential barriers and facilitators to intervention delivery. Parents and community members shared their preferences regarding the number and length of intervention sessions, mode of delivery (online vs. in‐person), format (group vs. individual), interventionist training protocols, and the importance of having ethnically and racially matched interventionists. Most participants expressed interest in a brief, online program led by interventionists who are fluent in their native language and who shared similar lived experiences.

Drawing from these insights, as well as prior literature on family‐based interventions with minoritized populations (Dariotis et al. 2008; Smith et al. 2018), we selected program features designed to enhance engagement and scalability. To address common barriers such as time constraints and transportation (Dumas et al. 2007), we chose a virtual, community‐based delivery format. We also prioritized recruiting interventionists who were community health professionals or educators and had experience working with parents and children (Lupini et al. 2025). To further promote feasibility and eventual scalability, we developed a training model that emphasized ease of use. After each session, interventionists self‐report on their fidelity and coverage of session topics (Sanders et al. 2020). This approach reduces reliance on external fidelity monitors and allows interventionists the opportunity to reflect on their performance.

The final step of IM is creating an evaluation plan. We applied a hybrid type 1 effectiveness‐implementation approach to assess family health outcomes and key implementation outcomes, including feasibility, acceptability, fidelity, and reach. Specifically, we used two implementation frameworks, the RE‐AIM and CFIR 2.0, to inform our selection and assessment of implementation outcomes. Our feasibility benchmarks (progression criteria) in accordance with the traffic light approach (Mellor et al. 2023) were: feasibility of recruiting (go/proceed with RCT: recruit ≥ 60 families in 12 months, amend/proceed with changes: recruit 40–50 families in 12 months, stop/do not proceed unless changes are possible: recruit ≤ 30 families in 12 months), feasibility of enrollment (go: ≥ 70% of eligible families enroll, amend: 40%–50%, stop: ≤ 30%), feasibility of session completion (go: ≥ 60% of randomized participants complete all six sessions, amend: 40%–50%, stop: ≤ 30%), and feasibility of assessment completion (go: ≥ 60% complete all three assessments, amend: 40%–50%, stop: ≤ 30%). Qualitative interviews with parents and interventionists will provide further evidence of feasibility, acceptability and other implementation outcomes intended to assess the conditions needed to support successful intervention delivery and dissemination. A full description of the evaluation plan is provided elsewhere (Cooper et al. 2025).

## 2 Discussion

This study demonstrates how IM can be applied and adapted to design culturally grounded, dual‐focused parenting interventions aimed at promoting the emotional and physical health of young Black and Latiné children. Using IM as a framework (Fernandez et al. 2019), we developed ROOTS, a brief universal prevention program that integrates positive parenting, ethnic‐racial socialization, and healthy lifestyle promotion. This work reflects key principles of community psychology, including an ecological focus on multilevel determinants of health, a strengths‐based orientation, and attention to structural inequities shaping child and family well‐being (Kloos et al. 2021; Rappaport 1977). This discussion highlights three key contributions of our approach: (a) the incorporation of health equity and cultural frameworks to enhance alignment with minoritized families’ lived experiences, (b) the integration of emotional and physical health within a unified model by identifying shared parenting processes that influence both domains, and (c) the embedding of implementation planning within early‐stage development to enhance real‐world feasibility and scalability.

### 2.1 The Value of IM for Culturally Relevant Intervention Design

Although IM does not explicitly embed cultural frameworks within each of the prescribed steps, we strategically leveraged its flexible structure to center cultural factors throughout program development. This intentional approach was essential to designing an intervention that reflected the lived experiences of Black and Latiné families. The first IM steps identified behavioral and environmental determinants influencing Black and Latiné children's health, such as cultural stressors (e.g., structural and interpersonal racism) and cultural strengths (e.g., ethnic‐racial socialization). Incorporating these factors into our logic models ensured that culture was positioned as a core driver of health outcomes. One important challenge involved identifying determinants shared across Black and Latiné families, with attention to their often distinct histories, traditions, and sociocultural contexts. We applied the Ecological Validity Model (Bernal et al. 1995) to guide decisions about whether and how to adapt intervention content across dimensions such as language, persons, metaphors, and delivery methods. This framework was particularly valuable for determining when cultural distinctions warranted unique tailoring (e.g., addressing deportation‐related fears among some Latiné families) versus when shared protective processes (e.g., positive parenting strategies like positive reinforcement) could be similarly emphasized across groups. Using the Ecological Validity Model within IM represents a practical strategy for integrating cultural adaptation frameworks into the early stages of intervention development.

This approach expands upon prior IM applications with ethnically and racially diverse populations, which, despite growing attention to cultural adaptation in prevention science (e.g., Barrera et al. 2017), have often relied on *surface‐level* adaptations (e.g., translated materials, incorporating culturally relevant images) rather than integrating cultural factors into intervention theories or logic models of change (Perry et al. 2017; Taylor et al. 2013). In contrast, our approach directly addressed health equity and implemented *deep structure* adaptations that positioned cultural strengths as active ingredients for health promotion. It is important to note that IM's framework requires researchers to actively prioritize equity and cultural considerations, as these do not automatically emerge from the prescribed steps. Our integration of cultural strengths and frameworks reflected our team's deliberate commitment to health equity. This concern is particularly salient given ongoing critiques of prevention science (Murry et al. 2024). Although the field has increasingly emphasized the importance of specifying for whom interventions are effective and under what conditions (Gottfredson et al. 2015), a substantial portion of the literature has not achieved these aims. Much of prevention research remains equity‐implicit or “one‐size‐fits‐all,” with limited attention to the role of structural racism (Buckley et al. 2025; Goodrum et al. 2024; Kerns et al. 2024), potentially contributing to gaps in adoption and effectiveness among minoritized populations (Butler and Titus 2015; Hall et al. 2016). Indeed, a recent review of 240 evidence‐based preventive interventions concluded that few demonstrated promise for reducing health disparities or could be considered equity‐promoting by their specified criteria (Buckley et al. 2025). Without intentional efforts to center equity and cultural responsiveness, IM applications risk reproducing these patterns. As such, we reconceptualized the “Logic Model of the Problem” as a “Logic Model of Child Health,” shifting from deficit framing toward a strength‐based lens, consistent with principles of community psychology (Rappaport 1977). This reframing has several advantages: it shifts how researchers engage with community partners (focusing on assets rather than deficits alone), shapes which determinants are prioritized (emphasizing protective factors alongside risk factors), and ultimately produces intervention content that is more culturally responsive and strengths‐based.

The integration of ethnic‐racial socialization into positive parenting represents a distinctive contribution of the ROOTS program and an advancement of the IM literature. Our scoping review of 31 dual‐focused parenting interventions found that few incorporated cultural strengths and none included ethnic‐racial socialization (Cooper et al. 2025). This is a notable gap, as research suggests ethnic‐racial socialization may buffer the effects of discrimination and promote positive identity development and health outcomes among children from minoritized backgrounds (Brody et al. 2021; Granberg et al. 2009; Neblett et al. 2008; Umaña‐Taylor and Hill 2020; Wang et al. 2022). The integration of cultural protective factors into a structured planning process outlines how IM can be leveraged to develop culturally grounded, strengths‐based prevention programs for minoritized families. While community psychology has long emphasized ecological perspectives, strengths‐based approaches, and attention to structural inequities, there remains limited guidance on how to systematically translate these principles into scalable interventions. The present study contributes to this gap by illustrating how such principles can be operationalized within a structured intervention development framework.

### 2.2 Bridging Emotional and Physical Domains

A second key contribution of this study was demonstrating how IM addresses two typically siloed health domains. The IM framework introduced a systematic method for identifying shared determinants of child emotional and physical health (e.g., family health routines, parental warmth and communication skills, and teaching cultural strengths). Recognizing these overlaps allowed us to streamline ROOTS into a cohesive program that addressed multiple aspects of child health without increasing intervention burden. Previous IM applications in early childhood have largely focused on single health domains, either healthy lifestyles and obesity prevention (Ball et al. 2017; Stea et al. 2016; Taylor et al. 2013), or social‐emotional outcomes (Blewitt et al. 2020; O'Connor et al. 2019). A small number of studies have begun to span both domains, such as programs targeting child self‐regulation in combination with healthy lifestyle behaviors (Ray et al. 2019; Tamblyn et al. 2025). However, virtually no prior IM‐based (or otherwise) early childhood interventions have achieved a strong and balanced dual focus, such as by targeting children's emotional health (e.g., prosocial behaviors, emotion regulation) and physical health (e.g., healthy lifestyle behaviors) through shared parenting mechanisms. ROOTS contributes to this emerging literature by demonstrating that IM can support the design of dual‐focused interventions that reflect the interconnected nature of child emotional and physical development.

### 2.3 Considering Implementation Issues From the Outset

A third contribution of this study was demonstrating how IM can incorporate implementation planning from the earliest stages of intervention development. Traditional non‐IM approaches often treat implementation as a future, long‐term goal to be addressed only after efficacy is established, which can lead to challenges with program adoption and scalability (Curran et al. 2012; Landes et al. 2019). In contrast, the IM process encouraged us to consider implementation determinants and strategies in parallel with the creation of program content. Early engagement with parents and community members identified potential barriers and facilitators to engagement and dissemination, such as time constraints, transportation issues, and a preference for culturally matched facilitators. This feedback directly shaped our design decisions, including the selection of a brief, online format and individually‐delivered sessions adaptable to families’ schedules. These choices were intended to increase acceptability and scalability potential. However, this efficiency also required substantial trade‐offs. Our logic models identified numerous behavioral and environmental determinants influencing child health. Addressing all of them comprehensively would require a longer, more intensive program. We prioritized core shared determinants of emotional and physical health (e.g., positive parenting, health‐promoting behaviors), which streamlined content but meant less intensive coverage of certain topics. For example, while ROOTS addresses healthy sleep and physical activity, it provides less depth than many sleep interventions or obesity prevention programs (Dawson‐McClure et al. 2014; Kidwell et al. 2019; Lumeng et al. 2017; Sepúlveda et al. 2020). This design choice reflects a deliberate prioritization of broad reach and scalability over depth of intervention for any single health domain. Evidence also suggests that simpler, lower‐intensity parenting interventions may yield outcomes comparable or even superior to longer, more complex programs, particularly when they involve universal prevention (Leijten et al. 2019). Future empirical testing will help determine whether this decision, favoring a brief approach, represents an appropriate trade‐off.

### 2.4 Strengths and Limitations

Overall, there were numerous advantages to using IM to develop a culturally relevant, dual‐focused parenting program. These included providing a systematic, transparent approach that integrated theory, research, and community input while also creating logic models and anticipating implementation challenges. Although some researchers have critiqued the intensity of the IM process, we believe these structured steps saved time, resulting in an intervention that is more likely to be successful, require fewer modifications at later stages, and that is adopted and sustained in real‐world settings.

Apart from these strengths, several limitations to our application of IM warrant acknowledgment. First, IM does not explicitly center equity. If researchers do not directly prioritize health equity and cultural responsiveness, these considerations may not naturally emerge from standard IM tasks. Second, traditional IM framing around “the health problem” may pose challenges for strength‐based approaches when working with marginalized communities. Our reconceptualization attempted to address this concern but represents an adaptation that researchers must intentionally implement. Third, although community voice informed all IM phases, community collaborators did not directly participate in all decisions in real time. Future applications could integrate a more formal community‐based participatory approach that promotes shared decision‐making at each stage of development, and that is more consistent with the traditional IM approach (Fernandez et al. 2019). Finally, empirical testing of ROOTS’ effectiveness, mechanisms of action, and scalability potential is necessary to determine whether our IM approach produced an intervention with substantial public health relevance.

### 2.5 Conclusion

This study suggests that IM, when adapted with concerted attention to equity and cultural alignment, can inform the development of preventive interventions addressing health disparities among minoritized populations. Future applications may benefit from prioritizing equity from the outset, integrating surface and deep structure cultural adaptations, and maintaining transparency about design trade‐offs when balancing effectiveness and scalability. Testing ROOTS’ effectiveness will provide important validation for this structured and culturally grounded approach for intervention development.

## Author Contributions

Daniel K. Cooper designed the study aims and methodology, contributed to all IM steps, and took the lead role in writing this manuscript. All authors (Daniel K. Cooper, Francesca Lupini, Subina Saini, Guillermo Wippold, Jayxa K. Alonzo, Darha M. Ponder, and Ronald J. Prinz) contributed to writing sections of the manuscript and read and approved the final product.

## Ethics Approval and Consent

The qualitative needs assessment activities described in this manuscript were approved by the University of South Carolina Institutional Review Board (IRB #: Pro00120313). All participants provided informed consent prior to participation. No clinical trial or outcome data are reported in this paper; the manuscript focuses exclusively on intervention development using the Intervention Mapping framework.

## Conflicts of Interest

The authors declare no conflicts of interest.

## Data Availability

No datasets were generated or analyzed for the current manuscript, which focuses on intervention development using the Intervention Mapping framework. Qualitative insights referenced from the needs assessment are summarized in aggregate to protect participant confidentiality.
